# New ways of working: COVID-19 as a catalyst for change in acute mental health services

**DOI:** 10.1192/j.eurpsy.2023.690

**Published:** 2023-07-19

**Authors:** K. Tong, G. Crudden, W. X. Tang, D. McGuiness, M. O’Grady, A. M. Doherty

**Affiliations:** ^1^National Forensic Mental Health Service, Central Mental Hospital, Dundrum; ^2^Department of Psychiatry, School of Medicine & Medical Science, University College Dublin; ^3^Department of Psychiatry, Mater Misericordiae University Hospital, Dublin, Ireland; ^4^East Sussex NHS Trust, East Sussex, United Kingdom; ^5^Department of Psychiatry, University Hospital Galway, Galway, Ireland

## Abstract

**Introduction:**

A need arose to divert patients with psychiatric complaints from the emergency department to alternative settings for psychiatric assessments to reduce footfall and to conduct consultations in a timely manner during COVID-19.

**Objectives:**

We assessed the effectiveness of alternative referral pathway in reducing COVID-19 infection in our service, and its effect on service quality: response time and number of patients leaving before review. We evaluated the satisfaction of patients, General Practitioners (GPs) and mental health service (MHS) staff with the pathway.

**Methods:**

All patients referred to the mental health service over a 2-month period following the introduction of the pathway were included. Findings were compared against the cohort referred for emergency assessment during the same period in 2019. Feedback surveys were distributed to patients, staff and GPs. χ ² and independent sample t-test were used to compare the variables.

**Results:**

Over 2 months, 255 patients received an emergency assessment via the pathway, representing a 22.3% decrease in the volume of presentations from the same period in 2019. There were no COVID-19 cases among our patients or staff on the roster for assessing patients. In comparison to 2019, response times were improved (p<0.001), and the numbers of patients who left the hospital before the review were reduced by 3.2% during the study period (p<0.001). Patients and GPs were highly satisfied with the referral pathway and believed that the pathway should be retained post-COVID-19. Mental health service staff were divided in their opinions about its sustainability.

**Conclusions:**

The pathway was successful in reducing the spread of infection, improving response times and reducing the numbers of patients who left without an assessment. Given the improved outcomes and acceptability, this is a preferable pathway for emergency referrals into the future.

**Disclosure of Interest:**

None Declared

